# Alkalinity cycling and carbonate chemistry decoupling in seagrass mystify processes of acidification mitigation

**DOI:** 10.1038/s41598-021-92771-2

**Published:** 2021-06-29

**Authors:** Cale A. Miller, Amanda L. Kelley

**Affiliations:** 1 Department of Evolution and Ecology, University of California Davis, Davis, CA 95616 USA; 2 College of Fisheries and Ocean Sciences, University of Alaska Fairbanks, Fairbanks, AK 99775 USA

**Keywords:** Biogeochemistry, Ecology, Ocean sciences

## Abstract

The adverse conditions of acidification on sensitive marine organisms have led to the investigation of bioremediation methods as a way to abate local acidification. This phytoremediation, by macrophytes, is expected to reduce the severity of acidification in nearshore habitats on short timescales. Characterizing the efficacy of phytoremediation can be challenging as residence time, tidal mixing, freshwater input, and a limited capacity to fully constrain the carbonate system can lead to erroneous conclusions. Here, we present in situ observations of carbonate chemistry relationships to seagrass habitats by comparing dense (DG), patchy (PG), and no grass (NG) *Zostera marina* pools in the high intertidal experiencing intermittent flooding. High-frequency measurements of pH, alkalinity (TA), and total-CO_2_ elucidate extreme diel cyclicity in all parameters. The DG pool displayed frequent decoupling between pH and aragonite saturation state (Ω_arg_) suggesting pH-based inferences of acidification remediation by seagrass can be misinterpreted as pH and Ω_arg_ can be independent stressors for some bivalves. Estimates show the DG pool had an integrated ΔTA of 550 μmol kg^−1^ over a 12 h period, which is ~ 60% > the PG and NG pools. We conclude habitats with mixed photosynthesizers (i.e., PG pool) result in less decoupling between pH and Ω_arg_.

## Introduction

The myriad biophysical factors that modify estuarine carbonate chemistry often transcend the effects of atmospheric CO_2_ hydrolysis in seawater (ocean acidification). These include the effects of groundwater flux, fluvial inputs, enhanced biological metabolism, eutrophication, upwelling, and tidal pumping, which interact in complexity resulting in coastal acidification^1–4^. The synthesis of these processes is a long-term pH variability that is estimated to be ~ 20 × greater than the open-ocean, where the increasing baseline of dissolved CO_2_ magnifies the frequency and duration of carbonate chemistry extremes resulting in impeded growth and development of calcifying organisms^1,5–9^. The deleterious socioeconomic implications of acidification has led to policy initiatives aimed at utilizing phytoremediation (i.e., photosynthetic CO_2_ uptake) by seagrass and kelp to locally mitigate acidification events^10–12^. This assumes that photosynthesis by macrophytes can reduce the dissolved inorganic carbon (TCO_2_)—simultaneously raising pH—during daylight hours when photosynthetic rates are high relative to heterotrophic respiration in seagrass beds and kelp forests. Initial research on this topic found that daytime reduction of TCO_2_ by seagrass is capable of increasing pH on short time scales, however residence time, depth, and enhanced community metabolism in nearshore seagrass habitats were found to dampen mitigation or exacerbate extreme conditions offering only minor, temporary, refuge from acidification^9,13–17^. These equivocal conclusions of carbonate chemistry variability in seagrass habitats may partially explain the contrasting correlations between bivalve growth and proximity to seagrass patches^18,19^. Notwithstanding the emerging complexity of phytoremediation, it is clear that further studies are needed to investigate the habitat specificity as it relates to biological communities and the physicochemical oceanographic dynamics of seagrass and kelp ecosystems from an acidification context.

To quantify phytoremediation by seagrass the carbonate system needs to be properly constrained as organismal sensitivities to acidification are specific to individual parameters (e.g., pH and CaCO_3_ saturation state Ω)—acidification is a multi-stressor^20,21^. Complicating matters is the nuance of carbonate chemistry variability in coastal margins where the potential for specific parameters to diverge from co-varying positive correlations (i.e., pH and Ω) is high, a phenomenon referred to as a decoupling of the carbonate system^1,22^. Previous studies have examined seagrass phytoremediation via autonomous pH and O_2_ sensors complemented by periodic discrete sampling of TCO_2_ or TA (total alkalinity)^9,15,17,18,23^, however logistics including site accessibility, timing of tidal cycles, and ability to conduct high-frequency sampling often precludes properly constraining the carbonate system. This can lead to the potential for measurement-estimation discrepancies^24^, which can equivocate occurrences of carbonate parameter decoupling and quantification of phytoremediation.

To date, most phytoremediation analyses from observations and modelling rely heavily on positive correlations between pH and seagrass density as a means to identify the potential for acidification mitigation^13,16,17^. Conclusions regarding acidification amelioration are, thus, limited by assumptions of a coupled carbonate system and an opacity to detail the multi-stressor component. In this study, we characterize the carbonate chemistry of adjacent pools in the high intertidal defined as being dense, patchy, or devoid of seagrass (*Zostera marina*). We measured multiple carbonate chemistry parameters (pH, TCO_2_, and TA) in each pool over a 17-d period at high frequency to identify tidal and diurnal patterns of carbonate chemistry change as a function of the presence and abundance of seagrass. Our analysis highlights the frequency of carbonate chemistry decoupling of pH and Ω_arg_ as it relates to seagrass density and TA variability. If this decoupling is common in seagrass beds, then previous estimates of phytoremediation are potentially overestimated due to assumptions about positive correlations among parameters (e.g., pH, Ω_arg_) despite the use of a robust TA-salinity relationship in calculating carbonate chemistry variables.

## Results

### Timeseries of pool carbonate chemistry

**Figure 1:**
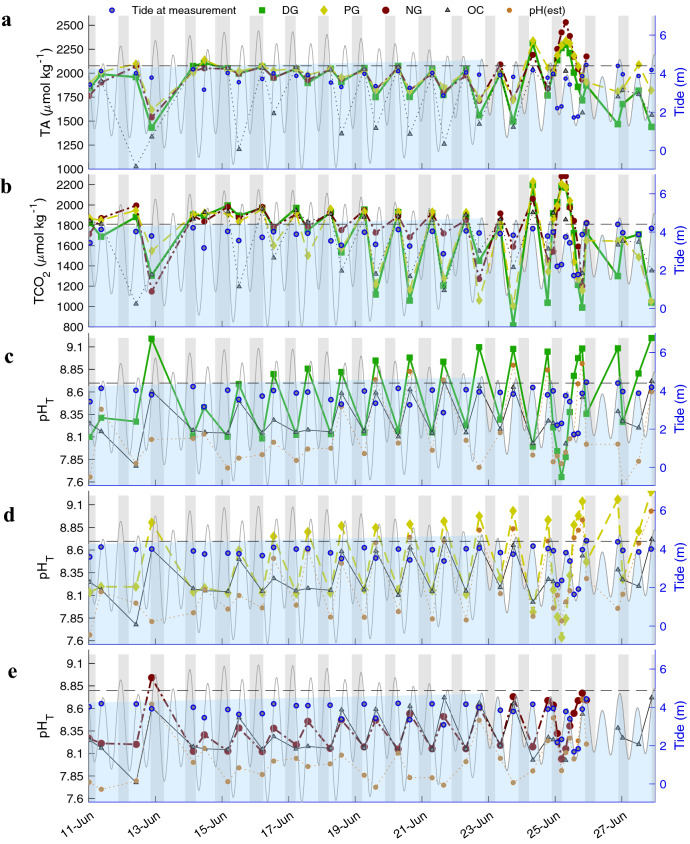
Timeseries of measured TA (**a**), TCO_2_ (**b**), pH_T_ and estimated pH_T_ (**c**–**e**) in dense grass (DG), patchy grass (PG), no grass (NG), and ocean (OC). Shaded blue region is predicted tide and shaded grey is night when PAR < 100 μmol photons m^−2^ s^−1^. Dashed line indicates tidal height when pools become immersed and tidal height at when samples were taken (blue dots). Three-hour sampling occurred for a period of 24 h starting 24 June 22:30.

Observations recorded in dense grass (DG: 62% of pool area), patchy grass (PG: 26% of pool area), and no grass (NG: 0% of pool area) pools at the head of Jakolof Bay, AK (Fig. S1), displayed robust hourly changes in TA, TCO_2_ and pH from 15–27 June (Fig. 1). An increasing dynamic range of ΔTA correlated with residence time, and to a more moderate degree, with ΔTCO_2_ (Fig. S2). Autocorrelation at lag of 2—corresponding to the troughs of the TA timeseries—was significant for the DG (*p* = 0.013), PG (*p* = 0.023), and NG (*p* = 0.002) pools. Immersion time (i.e., period pools were flooded) decreased from 3.85 to 2.60 h while depth of overlying water at high tide decreased from 1.18 to 0.30 m during the spring to neap tidal transition increasing the magnitude of carbonate chemistry change (Fig. 1). Over the 17-d period the ΔTA (μmol kg^−1^ h^−1^) range was greatest in the DG pool with a maximum value 43% greater than the PG pool, and 26% greater than the NG pool (Table S1). The ΔTCO_2_ maximum rate in the DG pool was double that of its ΔTA and only 23% and 48% greater than the PG and NG pools, respectively (Table S1). Immediately following each flood cycle when pools were emersed and retained an average depth of 6 cm, the TA, TCO_2_, and pH_T_ signals were approximately equal to ocean measurements and similar to normative estuarine systems. This is despite extremely low salinity (Fig. S3) at the surficial layer of the incoming flood tide which lowered ocean TA only at the surface. Small fluctuations in pool salinity (≤ 1), however, correlated well (slope = − 0.466; R^2^ = 0.849) with changes in depth (cm) which ranged between 1–2 cm when using the NG pool as reference.

Estimated pH_T_ calculated from TCO_2_ and TA in all pools were robustly different from direct pH_T_ measurements resulting in a measurement-estimation discrepancy (Fig. 1c–e). Measured values were consistently higher where the mean of pH_T_–pH_T_est_ (± SD) was 0.38 ± 0.35 for DG, 0.22 ± 0.26 for PG, and 0.33 ± 0.23 for NG pools. The NG pool flooded at a height 0.23 m higher than DG and PG pools resulting in an extended emersion period and complete evaporation of the pool on 26 June producing anomalous measurements after the 24th as robust deviations became present (Fig. S3). The PG pool had the greatest overall pH_T_ range from 7.64 to 9.25 while the daily extremes were slightly less from 7.64 to 9.14, and 7.65 to 9.08 for the DG and NG pool, respectively. The daily increase in pH_T_ occurred concomitantly with temperature that ranged from ~ 12.4 to 26.6 (Fig. S3), which would thermodynamically decrease pH_T_ by ~ 0.225 units over the TA and salinity values observed due to the positive correlation between temperature and carbonic acid dissociation constants.

### Hourly rates of change during emersion

**Figure 2:**
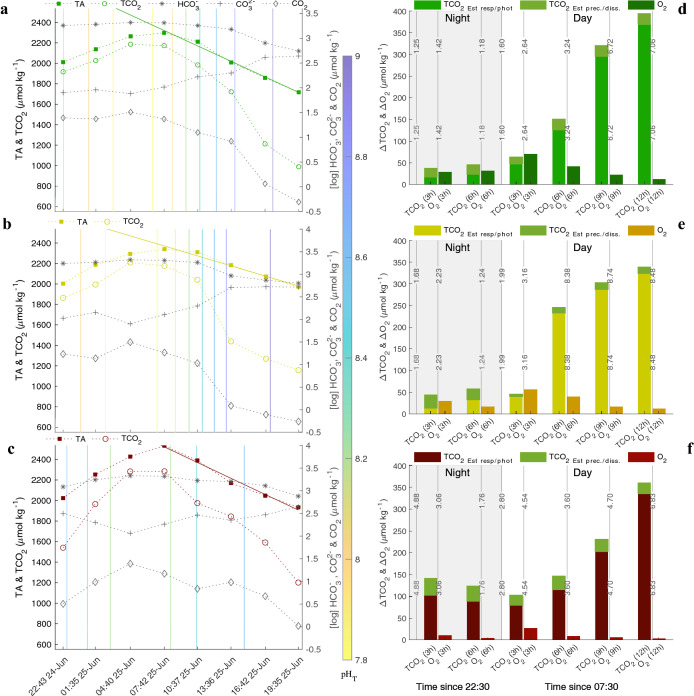
Alkalinity and TCO_2_ (left y-axis) during 21 h emersion sampling period for dense grass (**a**), patchy grass (**b**), and no grass (**c**) pools. The log concentration of CO_2_, HCO_3_^-^, and CO_3_^2-^ are marked as grey on the right y-axis with colored pH_T_ isoclines. Absolute values of ΔTCO_2_ and ΔO_2_ during same emersion period where the total ΔTCO_2_ was estimated based on proportion of change due to biological respiration/photosynthesis or CaCO_3_ precipitation/dissolution for dense grass (**d**), patchy grass (**e**), and no grass (**f**). Isoclines are Ω_arg_. Note: The NG pool during this period began to experience increased salinity due to evaporation reducing confidence in the displayed values.

High-frequency sampling of carbonate chemistry and ancillary parameters (O_2_, temperature, salinity, and nutrients) characterize a diel modulation for all pools with increases in TA and TCO_2_ at night (PAR < 100 μmol photons m^−2^ s^−1^) and decreases during the day (Fig. 2a–c). Hourly nighttime increases in TA and TCO_2_ for DG and PG pools were 42.13 and 48.50 (R^2^ = 0.99 and 0.98), and 44.99 and 173.08 (R^2^ = 0.99 and 0.92) μmol kg^−1^ h^−1^, respectively. The ratio at which TA and TCO_2_ increased for the DG (1.07) and PG (1.14) pools was ~ 1 whereas the NG pool had a TA:TCO_2_ ratio of 1.84. In daytime, TA decreased linearly for all pools at a rate ~ 51.0 μmol kg^−1^ h^−1^ (R^2^ = 0.98) for DG and NG pools, and 32.3 (R^2^ = 0.97) μmol kg^−1^ h^−1^ for the PG pool. TCO_2_ decreased fastest in the PG pool resulting in higher pH_T_ earlier in the day compared to DG and NG pools, and a more rapid shift in carbonate chemistry speciation from HCO_3_^−^ to CO_3_^2−^. Supersaturation of CaCO_3_ (Ω_arg_) persisted in all pools for the entire emersion period (~ 21 h) regardless of PAR levels (Fig. 2d–f). Estimates of TCO_2_ modification attributable to photosynthesis and respiration as well as CaCO_3_ precipitation or dissolution—estimated from changes in TA—show that respiration and photosynthesis was the predominate mechanism of ΔTCO_2_. The degree of ΔTCO_2_ appeared to far outpace ΔO_2_, which peaked in late morning.

### Alkalinity drawdown and associations with seagrass

**Figure 3:**
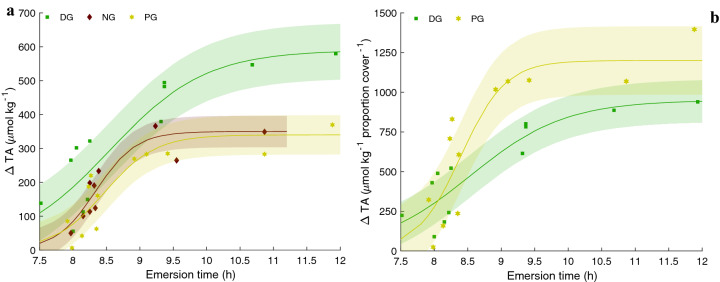
The ΔTA (**a**) and ΔTA per proportion of seagrass cover (**b**) as a function of emersion time for dense grass (DG), patchy grass (PG), and no grass (NG) pools. Highlighted region is the RMSE of modeled logistical fit.

Logistical curve fits to ΔTA as a function of emersion time was greatest in the DG pool reaching a maximum of ~ 550 μmol kg^−1^ (RMSE = 82.3) around 10.5 h compared to the NG (RMSE = 46.4) and PG (RMSE = 58.0) pools with ΔTA maxima of ~ 300 μmol kg^−1^ at 8.5 h (Fig. 3a). At maximum ΔTA, DG was significantly different than both PG and NG pools represented by nonoverlapping model bounds. The ΔTA per proportion of seagrass cover over the same emersion period appeared greater in the PG pool relative to the DG pool, however, the large RMSE (DG = 133.3 and PG = 215.5) bounds suggests these two pools are undistinguishable (Fig. 3b).

### Carbonate chemistry decoupling and estimate discrepancies

**Figure 4:**
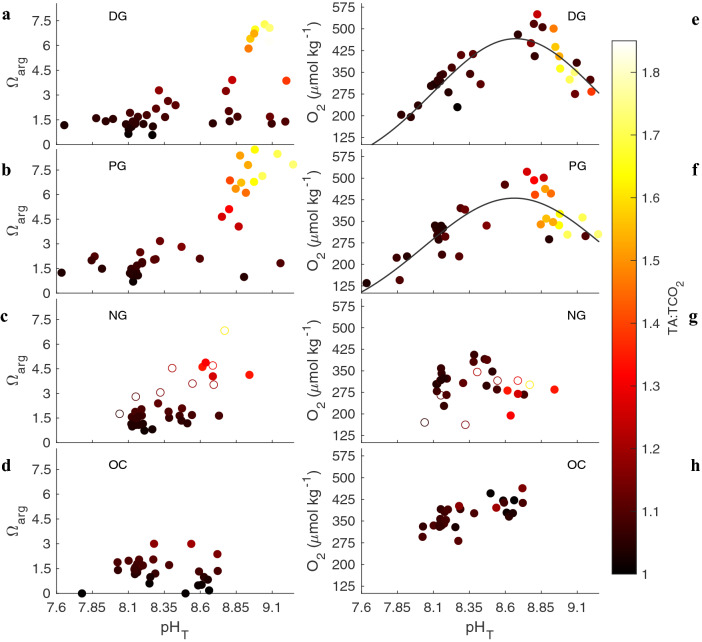
All sample points of Ω_arg_ (**a**–**d**) and O_2_ concentration (**e–h**) as a function of pH_T_ for dense grass (DG), patchy grass (PG), no grass (NG), and ocean (OC). Color bar is the measured TA:TCO_2_ ratio for each point in the timeseries. Gaussian fits applied to DG pool with an RMSE of 51.39 and 55.68 for the PG pool. Open circles in panels (**c**) and (**g**) indicate measurements taken after 24 June during NG pool evaporation.

Aragonite saturation state and [O_2_] relationships with pH_T_ varied by pool for the entire timeseries. A decoupling from a consistent positive correlation between pH_T_ and Ω_arg_ was observed in the DG and PG pools which displayed Ω_arg_ < 1.5 across a pH_T_ range 7.63–9.10, with a greater proportion of low Ω_arg_ values at high pH_T_ in the DG pool relative to the PG pool (Fig. 4). The [O_2_] in the DG and PG pools followed a gaussian distribution with an RMSE of 51.4 and 55.7, respectively, where [O_2_] peaked at a pH_T_ ~ 8.8 and then began to decrease. At this pH_T_ and associated TA:TCO_2_ ratio, the speciation of TCO_2_ becomes approximately equal between HCO_3_^−^ and CO_3_^2−^ concentrations. The trend of O_2_ decrease at this threshold was not present in the NG pool, and a linear relationship was observed in the ocean signal.

**Figure 5:**
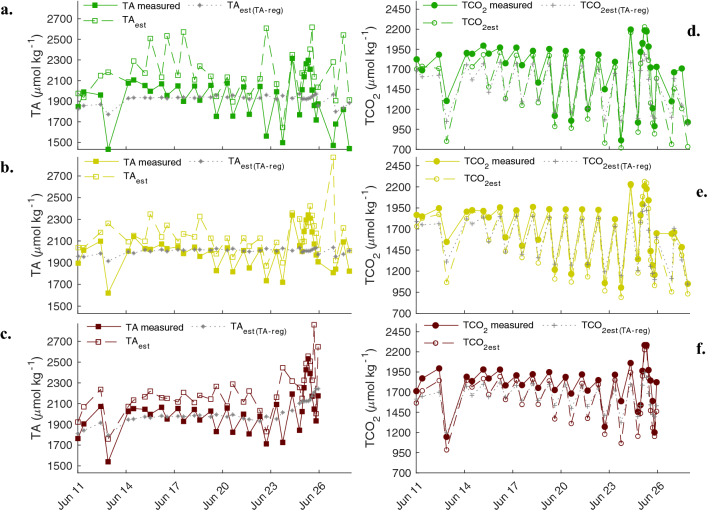
Measured TA (closed squares) along with TA estimated from pH_T_ and TCO_2_ (open squares) and TA-salinity regression (grey *) for the DG (**a**), PG (**b**), and NG (**c**), pools. TCO_2_ measured values (closed circles) along with TCO_2_ estimated from pH_T_ and TA (open circles) and from pH_T_ and TA derived from TA-salinity regression (grey +) for DG (**d**), PG (**e**), and NG (**f**) pools.

Measured TA and TCO_2_ deviated from estimated values when using two auxiliary carbonate system variables, corroborating decoupling between pH_T_ and Ω_arg_ in all pools. Estimated TA was predominately greater in all pools ranging from 1049 to − 53, 1063 to −  92, and 813 to 18 μmol kg^−1^ in the DG, PG, and NG pools, respectively (Fig. 5). The converse was true for TCO_2_ where measured values were majority greater than estimated values. The TA estimates derived from the TA-salinity regression were modest relative to measured values, with ranges below 200 μmol kg^−1^ for the DG and PG pools, and ~ 450 μmol kg^−1^ for the NG pool (Fig. 5). We note that the R^2^ was poor for the DG and PG pools and only moderate in the NG pool (Fig. S4). TCO_2_ derived values calculated from an estimated TA based on regression with salinity approximately followed TCO_2_ estimated from measured pH_T_ and TA. Discrepancies as great as ~ 250 μmol kg^−1^, however, were still observed (Fig. 5). For all estimated values, the timepoints that are most congruent with actual measured values occur at the peaks of the timeseries. These were periods immediately following the flood tide where the more homogenous oceanic signal replaced local pool carbonate chemistry dynamics.

## Discussion

The extreme diel cyclicity of TA observed in this study is unprecedented for nearshore seagrass habitats in temperate locations and refutes assumptions of its invariability and strong correlation with salinity, a relationship often used to constrain the carbonate system. The modulation of pH_T_, TCO_2_, and TA in each of the pools exceeds those that would be derived based on any two of the carbonate chemistry parameters resulting in measurement-estimation discrepancies and an inability to accurately quantify a decoupled system—a situation likely overlooked by previous studies^15–17,23,25^. Extreme decoupling of the carbonate system was present in the DG pool only, where pH_T_ ranged from 7.65 to 9.19 while maintaining a salinity > 26 and Ω_arg_ < 1.5—a threshold at which acute stress occurs in certain bivalve larva^26^. The two seagrass pools (DG, PG) displayed fundamental differences as it relates to extremes in carbonate chemistry variability, characteristic of previous findings detailing an exacerbation of extremes in seagrass habitats^9^. Despite abundant filamentous macroalgae and observed microphytobenthos (Fig. S1), the NG pool exhibited a reduced magnitude of variability and maintained a mostly positive correlation between pH_T_ and Ω_arg_. The OC signal experienced more modest decoupling, however this was due to the freshwater lens at the surface upon the incoming flood tide. While phytoremediation may appear present during occasions with extremely high pH_T_ and Ω_arg_, the reduction of TCO_2_ and bioavailable carbon for calcification remained extremely low, potentially impeding organismal calcification^27–29^, inducing a result opposite of phytoremediation. While these conclusions are based on in situ timeseries sampling without replication, the consistent behavior and difference between each pool and autocorrelation over the timeseries gives confidence in our conclusions.

**Figure 6:**
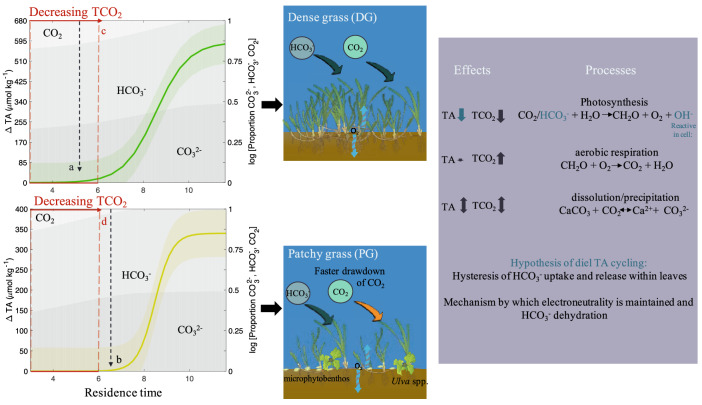
Conceptual model plots of ΔTA as a function of residence time and the concomitant change in carbonate chemistry speciation (shaded in grey) for dense grass (green) and patchy grass (yellow) based on measurements presented in Figs. 3 and 4. Dashed lines on plots indicate specific time points at which TA decreases: (**a**) TA begins to decrease at 5 h when [CO_2_] is still moderate, (**b**) TA begins to decrease at 6.5 h when [CO_2_] is nearly exhausted, (**c**) 3–6 h residence time TCO_2_ decreases at rate of ~ − 85 µmol kg^−1^ h^−1^, and (**d**) 3–6 h residence time TCO_2_ decreases at rate of − 205 µmol kg^−1^ h^−1^. Stoichiometric equations and the effects on TA are shown in purple box with hypothesized processes modulating TA based on this study in dark blue text.

Model estimates of daytime ΔTA as a function of residence time suggest that the mixed autotroph PG pool resulted in a lower integrated TA decrease but a faster rate of TCO_2_ drawdown shifting the distribution of carbonate chemistry speciation to limited CO_2_ availability earlier in time (Fig. 6). Based on our results, we hypothesize that higher photosynthetic rates by non-seagrass photosynthesizers (e.g., microphytobenthos) in mixed seagrass communities can raise pH and drawdown TCO_2_ faster leading to a more rapid increase in TA:TCO_2_ due to the TCO_2_ uptake physiology by those autotrophs^14,30,31^. This is counter to other assertions suggesting greater seagrass density (leaf area index) leads to a greater potential of acidification remediation^13^; it is clear though, that a more rigorous characterization of the carbonate system is needed to address the efficacy of mitigation and potential decoupling of the system as demonstrated by this study.

The effects of extreme TA diel cycling modify the acid–base chemistry and, thus, the sensitivity of Ω_arg_, pH, and PCO_2_ to subsequent fluctuations in TA and TCO_2_^32^. The TA:TCO_2_ ratio ranged from 1.05 to 1.80 with highs correlated to longer residence times during neap tidal periods that affected the duration of immersion and emersion. Studies that have previously recorded such changes in TA identify benthic flux or CaCO_3_ dissolution as the driver of diel variability, however the attributes of the sediment in those habitats were permeable, medium sand or coarse, and rich in CaCO_3_—conducive conditions for enhanced diffusive and advective efflux from porewater^33–35^. While decreases in TA can be a result of calcification by epibionts or seagrass leaves themselves, those instances occurred in tropical environments with waters extremely high in CaCO_3_, or in temperate nearshore waters where riverine inputs carried high [TCO2] and [TA], and Ω_arg_ > 10 in the submerged aquatic habitats; however, neither reported diel cycling of TA or found the unique TA:TCO_2_ ratios observed here^36,37^. In this study site, the sediment in the study pools was comprised of slate and greywacke, comingled with densely packed fine-grained clay and silt, attenuating in-sediment permeability. Porewater profiles at 1, 2, and 3 cm depths depicted [TCO_2_] > [TA_Carb_] and were consistently higher than the concentrations in the overlying water (Fig. S5). Given this orientation, efflux of TA and TCO_2_ from porewater should be persistent, particularly as the concentration gradient would increase during the day as TA and TCO_2_ decreased in the pools.

If we assume the solute exchange between porewater and overlying water was the mechanism for observed diel TA variability, we can estimate an integrated benthic flux at night in the DG pool of 4.4 mmol m^−2^ (~ 6 h) and ~ 10 mmol m^−2^ (~ 12 h) during the day, with daytime rates for the PG and NG pools slightly lower at ~ 5.5 mmol m^−2^. These rate estimates are similar to those reported in tropical environments where TA flux occurs concomitantly with dissolution in permeable sediments^33^. There was no evidence of high CaCO_3_ in the muddy sediments at this site, however, nor of calcifying epiphytes as this region is dominated by cyanobacteria and diatoms at the sediment surface^38^—epiphytic growth, overall, was surprisingly minimal. The linear changes in TA over the 21 h period would assume a diffusive flux with a likely stagnant boundary layer because advective processes that can enhance flux rates were limited to bio-irrigation and- turbation—which visually appeared minimal—due to lack of other forces (e.g., wave, current, tide). The characteristics of TA variability were expressed as linear rates of change during the day and at night, whereas TCO_2_ changes were nonlinear during the day and linear at night. At night TA and TCO_2_ increased ~ 1:1 in the DG and PG pools, which exemplified a possible scenario of CaCO_3_ dissolution (2:1 change) coupled with respiration that would change TA:TCO_2_ 0:1. If plausible, this would have to occur in superstrated waters (Ω_arg_ was > 1) where the ΔO_2_ roughly matches the estimated—respiration only—ΔTCO_2_. This was not the case, however, as the ΔTCO_2_ was < ΔO_2_ after 3 h (ratio of 0.55 and 0.44, respectively) and mixed after 6 h with a ratio of 0.75 and 2.0 in the DG and PG pools, respectively. It is possible denitrification-nitrification processes could account for this excess O_2_ by lowering the community respiration quotient as has been seen in seagrass communities^39^. While we note that biogenic CaCO_3_ dissolution occurring at Ω_arg_ > 1 is possible, the rate of dissolution is minimal in supersaturated waters and dependent on specific polymorphs^40^. Additionally, the rate of ΔTA is consistent despite the temperature fluctuation in the pools, which would enhance the rate of dissolution increasing the rate of TA change: this was not observed. These speculations for TA variability of course assume ΔTCO_2_ could be partitioned by precipitation/dissolution, of which there is no strong evidence.

Nutrient assimilation and remineralization as well as organic alkalinity can also contribute to changes in TA^41–45^, but not at the scale of change observed here. Over the high frequency sampling period when nutrients were measured, the stochasticity and low magnitude of change in PO_4_^3−^, SiO_2_, NO_x_, and NH_4_^+^ were not found to correlate with ΔTA (Table S2). The changes in TA observed were 1–2 orders of magnitude greater than changes in nutrient concentrations resulting in a trivial addition to ΔTA. In addition, presence of organic alkalinity derived from phytoplankton or humic organic compounds would result in measured TA values > estimated values, but this was not the case. This further complicates the identification of processes responsible for TA variability and, thus, provokes the speculation of non-dissolution and nutrient cycling mechanisms.

The modulation of TA and TCO_2_ at a 1:1 ratio during night and a decreasing ratio during the day as carbon becomes potentially limiting for seagrass gives credence to HCO_3_^-^ as a potential source of TA cycling. Specific seagrass species including *Z. marina* and other macrophytes are known to utilize HCO_3_^-^ as a carbon source^46–48^. Evidence suggests the active uptake of HCO_3_^−^ occurs via H^+^ symport in *P. oceanica*, where electroneutrality is likely preserved by a Cl^−^ or NO_3_^−^ efflux and Na^+^/H^+^ antiport^49,50^. The accumulation of HCO_3_^-^ in *P. oceanica* aids in the establishment of a robust electronegative potential in the leaves, which is also a phenomenon present in *Z. marina*^50^. The accumulation of HCO_3_^-^ within the cell wall may occur at a faster rate than the dehydration to CO_2_ in the cytoplast catalyzed by carbonic anhydrase, which could lead to efflux of undehydrated HCO_3_^−^ at night when light energy is limiting. This could also explain the disparity between O_2_ generation and TCO_2_ drawdown during the day as hysteresis can occur on the timescale of hours^51^, which could be further modified by photorespiration^52^. The evidence here, along with the suggested mechanisms by which electroneutrality is preserved when HCO_3_^−^ uptake occurs could explain the decrease in TA observed in these pools when TCO_2_ is limiting. Photosynthesis, however, is not presumed to affect TA even if HCO_3_^−^ is utilized because it is expected that uptake would be compensated with H^+^ or OH^−^ exchange^43^. More evidence is needed though to determine if this is the case in higher order photosynthesizers because research shows electroneutrality preservation by OH^−^ and H^+^ may be replaced by NA^+^, Cl^−^ and NO_3_^−^ in some seagrasses^48,49,53^. Anomalous to this conclusion would be the observed TA variability in the NG pool. The *Ulva* spp., which was observed in the NG pool, however, can also take up HCO_3_^−^ via ion exchange, potentially with OH^-^ or Cl^−^^47,54^. While further investigation is needed to identify the mechanism of TA variability in these pools, similarly low TA values and cycling have been recorded in enclosed bays with long residence times and abundant seagrass, and even speculation of HCO_3_^-^ was noted as the potential driver of the observed TA variability^35,52,55^. These incidences of low TA and its diel cycling, however, were not assessed from a phytoremediation standpoint, thus the implications hitherto remained unappreciated.

The pools in this study are representative microcosms of larger systems, replicating carbonate chemistry variability in seagrass habitats on a magnified scale. Perched estuaries, lagoons, and enclosed bays with long residence times have recorded extremely high pH values similar to those found in this study, including instances of reduced TA: correlated to the proximity of oceanic influence^52,55,56^. In these systems, biological metabolism and evaporation become the dominate mechanisms that modify pH^5,56,57^. It would be remiss to note, however, that the thermodynamic controls on carbonate chemistry also become enhanced at long residence times as was seen in this study. Observed temperature fluctuations ranged from ~ 12.5–26.5 °C increasing the carbonic acid dissociation constants resulting in an estimated pH drop of ~ 0.21 units across the recorded pH and TA range (Fig. S2); however, this was superseded by photosynthetic modification of pH. Evaporation appeared to have little effect on the DG and PG pools with respect to salinity as values remained fairly constant even at extreme temperatures (Fig. S2). Noticeable was the complete evaporation of the NG pool and the rapid salinity increase, suggesting that small variations in the volume of tidal immersion (i.e., depth of overlying water) can modulate the degree to which estuary carbonate chemistry is affected by the slope of the tidal region.

Findings here show unequivocal, frequent, decoupling of carbonate chemistry that would otherwise be overlooked without properly constraining the system and absolving measurement-estimation discrepancies. This can lead to an overestimation of phytoremediation when conclusions are based on pH variability rather than the total carbonate acid–base chemistry system^17,23^. This can occur despite a robust TA-salinity relationship or because of a mischaracterization of TA variability if results are derived from discrete measurements based on specific sampling times corresponding to tidal period, proximity to macrophyte habitat (above or in canopy) and coupled with pH-only monitoring. In accordance with previous studies, findings here show that long residence times and spatial variability along with tidal and current mixing are strong drivers for determining carbonate chemistry variability in seagrass^13,15^, however, we show that decoupling of the carbonate system in dense seagrass occurs rapidly, is independent of low salinity, and is likely related to extreme TA variability potentially caused by a hysteresis of how HCO_3_^-^ cycles within the medium in a carbon-limited system.

## Materials and methods

### Site description and assessment

Jakolof Bay is located in the outer portion of Kachemak Bay where local oceanographic conditions and carbonate chemistry are driven by exogenous characteristics from the Gulf of Alaska and autochthonous biological metabolism^58^. Jakolof Bay is a small fjord (3.5 km in length) that opens up into Kasitsna Bay, fed terrestrially by Jakolof Creek which runs along the north and south boarding the elevated salt marsh and the higher tidal flat—interspersed with depressions—where study site pools were located. The topography of the site suggests that minimal terrestrial subterranean groundwater reaches the pools which are at elevation from the land side creek. Thus, porewater intrusion likely originates from the oceanic front during the floodtide pressure gradient. The geology of Jakolof Bay consists primarily of highly metamorphosed slate and graywacke, likely from the Triassic Period^59^. The sediment in each pool was muddy with interspersed slate gravel. The outer region of Jakolof Bay sediment is ~ 60% fine grained silt and clay^60^, a similar sediment characteristic to that found in the pools. Species characterization of the intertidal in Jakolof is scarce, but shallow subtidal assessments identified dominant taxa as Polychaetes, Malacostraca, Gastropods, and Bivalves, however, the diversity of these groups was fairly low relative to other fjords in the region and the deeper areas of Jakolof Bay^60^. Pacific blue mussels (*Mytilus trossulus*) were observed in the creek channels, while scattered amphipods and sparse *Littorina* spp. feeding on the fleshy macroalgae appeared to be the only potential calcifiers in the pools, which were transported in-and-out during flood and ebb. This was the extent of the fauna characterization, although presence appeared to be minimal as well as epiphytic growth on seagrass (Fig. S1).

### Sample collection and processing

Three shallow, adjacent pools with varying depth on the edges, within 50 m of one another were selected as sample sites and characterized as dense grass (DG), patchy grass (PG), and no grass (NG) with ellipse areas ~ 330.9, 180.0, 339.9 m^2^ with average center depths 5.1, 6.2, and 7.1 cm, respectively, in the high intertidal of Jakolof Bay, AK: 59°26′54.09″ N, 151°29′49.96″ W (Fig. S1). TA, TCO_2_, pH_T_, O_2_, and salinity samples were collected toward the center of pools at maximum depth at intervals ~ 16 and 8 h for 17 d, with high-frequency sampling occurring every 3 h on 24 June 22:30 AKT during a 21 h emersion period. TA, pH_T_ and O_2_ samples were collected in two separate 150 mL borosilicate bottles (one with optical dot), while measurements for TCO_2_ were collected in a 5 mL centrifugal tube and poisoned for preservation with 10 μL of saturated HgCl_2_. Temperature measurements were made in situ with an Omega HH81A digital thermometer, while TA, pH_T_, salinity, and O_2_, were measured at Kasitsna Bay Laboratory within 30 min of collection. TA was measured using an Apollo SciTech AS-ALK_2_ with duplicate titrations performed haphazardly throughout the entire sample collection (average duplicate uncertainty 6.71, SD ± 7.48), and CRMs (certified reference material: batch #181) measured before and after each machine calibrated run. pH_T_ was measured potentiometrically with a Thermo Scientific ROSS Ultra electrode calibrated at total scale with Tris buffer and corrected with an offset that was derived from a regression of 25 samples between potentiometric and spectrophotometric measurements using a Shimadzu UV-1900: this offset was 0.019 units (Fig. S5). O_2_ measurements were performed with a PreSens Fibox 4 (using factory calibration) after each sample was collected and immediately stored in a dark box in the field until measurement ~ 30 min later. Salinity was measured with a YSI 3100 conductivity meter. All TCO_2_ samples were run at Shannon Point Marine Center, WA, on an Apollo SciTech AS-C3 along with several CRMs (batch #179) interspersed after calibration. Each TCO_2_ measurement reported was the average of the three closest analytical measurements that were < 10 μmol kg^−1^ between each measurement. The uncertainty for measured TA (7.58 ± 8.78 SD) and TCO_2_ (6.77 ± 6.32 SD) was the average difference between known CRM and measured CRM across all samples.

Estimated pH_T_ and Ω_arg_ were calculated using CO2SYS (Matlab V1.1) with inputs TA and TCO_2_ using the carbonic acid dissociation constants from Lueker et al.^61^, the bisulfate dissociation constant of Dickson et al.^62^, and the boron constant from Uppström^63^. Assuming the ΔTCO_2_ over the 21 h sampling period was a response to photosynthesis/respiration and CaCO_3_ precipitation/dissolution, estimates of partitioned ΔTCO_2_ were calculated using the absolute hourly rate of change for TCO_2_ and TA during each time point. Where ΔTA*0.5 (based on the alkalinity anomaly) was equal to the ΔTCO_2_ as a result of precipitation or dissolution, and the remainder of ΔTCO2–ΔTA*0.5 was a due to biological metabolism. Proportion of cover was calculated by measuring the length and width of each pool and then using photographed images to define the proportion of pool area covered by *Z. marina* present as a ratio of pool size using ImageJ (v. 1.53a). Significance of autocorrelation lag points on TA timeseries for each pool was determined using a Ljung-Box Q-test.

### Model construction

A three-parameter logistic curve fitting routine was applied to each pool correlating ΔTA with emersion time when samples were collected immediately after and before flood tide. This ended up being 12 time points for DG and PG pools and 10 for the NG pool (pool evaporated at day 15) during the change in tidal cycle from spring to neap. A conceptual model of carbonate chemistry dynamics for the DG and PG pools was determined by using the estimated values of ΔTA across the entire time series (described above) and the changes in carbonate chemistry speciation derived from the high frequency 21 h sampling period. Since the ΔTA during the 21 h sampling period was integrated into the entire timeseries estimates, the carbonate speciation changes are representative of the expected dynamics that would be visible at other time points.

### Porewater sampling

Porewater collectors were assembled using Super Speedfit polypropylene 6.35 mm press-connect fittings. T-shaped connectors were fit with two, ~ 3.8 cm length pieces of food grade plastic tubing, with open ends sealed with thermoplastic hot melt adhesive. Two sides of each piece of tubing were punctured five times with a 16-gauge needle, wrapped with a 0.45 micron PES membrane filter and adhered with thermoplastic adhesive to keep out sediment. Three collectors for each pool were buried at 1, 2, and 3 cm depths (Fig. S1), ~ 36 h and 3 tidal cycles before the first samples were collected. The top of each T-connector—which protruded above the sediment—was sealed with a Super Speedfit polypropylene plug.

Prior to collection, 5 mL centrifugal tubes were placed in a glove bag purged of O_2_ and filled with N_2_. Vials sat in a glove bag with caps off for 1–2 h while being filled with N_2_ and shaken haphazardly. The caps of each vial had a 6.35 mm hole drilled in the top and then covered with electrical tape. Caps were secured from inside glove bag and vials then stored and transported in a plastic bag. A 10 mL serological pipette fitted with a 6.35 mm piece of rigid tubing was fit into the top of each press-connect securing a tight connection and porewater slowly extracted. The first 2 mL of water was discarded and 4–5 mL transferred to the vial by removing tape temporarily and injecting the collected porewater into the vial. Samples were measured immediate for pH_T_ in glove bag using a Thermo Scientific ROSS Ultra electrode calibrated with Tris buffer and reported on the total scale. A 0.019 correction factor derived from a 25-sample regression between potentiometric and spectrophotometric measurements using a Shimadzu UV-1900 was applied. After pH measurement, samples were poisoned with 10 μL of saturated HgCL_2_ and capped with a new screw top until TCO_2_ was measured at Shannon Point Marine Center, Anacortes, WA, on an Apollo SciTech AS-C3. Samples were collected every 3 d.

### Nutrient sampling

Nutrient NO_x_, NH^4+^, PO_4_^3−^, and SiO_4_ samples were collected in each pool every 3 h during the 21 h sampling period starting 24 June 2019. Water samples were collected with a 60 mL syringe and filtered through a GFF with a particle retention rate of 1.2 µm into a 20 mL scintillation vial. Samples were frozen at – 4 °C within 30 min of collection until analysis. Samples were processed on a SmartChem multi-element analyzer at Oregon State University.

## Supplementary Information

Supplementary Information.
