# Supplementary material for: Alkalinity cycling and carbonate chemistry decoupling in seagrass mystify processes of acidification mitigation
Source: Sci Rep. 2021 Jun 29;11:13500. doi: 10.1038/s41598-021-92771-2 (PMC8241997; doi:10.1038/s41598-021-92771-2)
Supplement: Supplementary file 1 — Supplementary Information. [file 41598_2021_92771_MOESM1_ESM.pdf]

Supplementary Information for
Alkalinity cycling and carbonate chemistry decoupling in seagrass mystify processes of acidification mitigation

Cale A. Miller and Amanda L. Kelley
Email: cmill@ucdavis.edu; calemiller620@gmail.com

Supplementary Figures and Tables

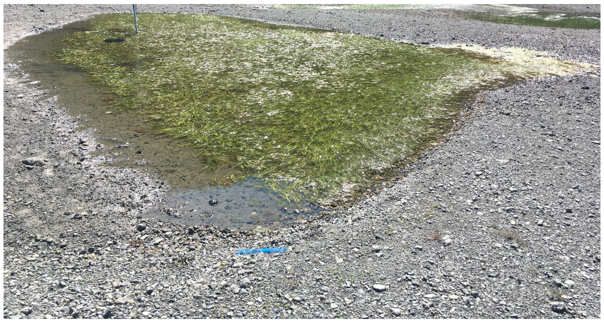

a. Dense grass (DG) pool

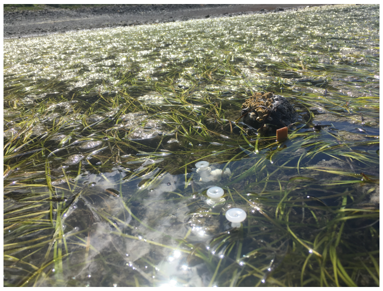

d. Porewater collectors in DG pool

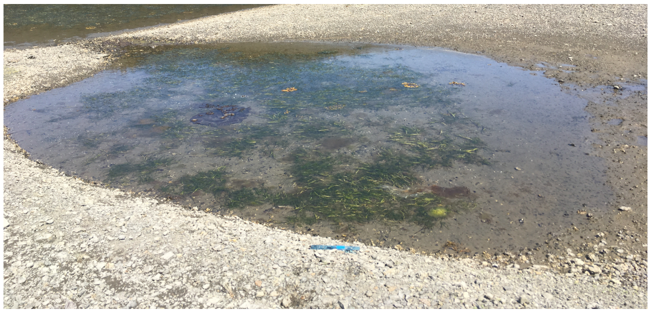

b. Patchy grass (PG) pool

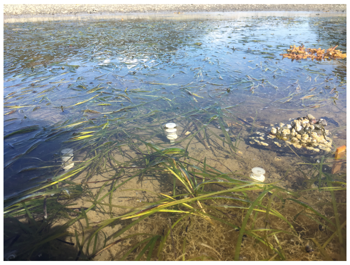

e. Porewater collectors in PG pool

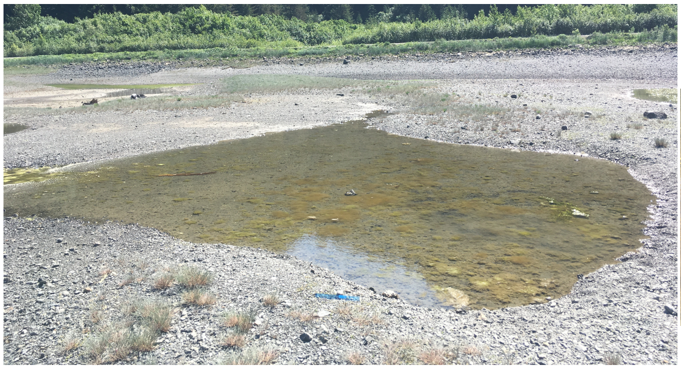

c. No grass (NG) pool

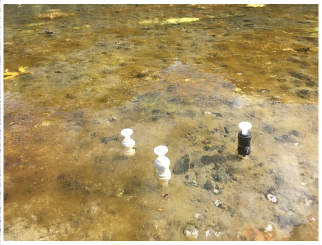

f. Porewater collectors in NG pool

Figure S1. Sample site of dense grass (a), patchy grass (b), and no grass (c) pools in Jakolof Bay, Alaska, USA. Porewater collectors placed at 1, 2, and 3 cm depths in the DG (d), PG, (e), and NG (f) pools.

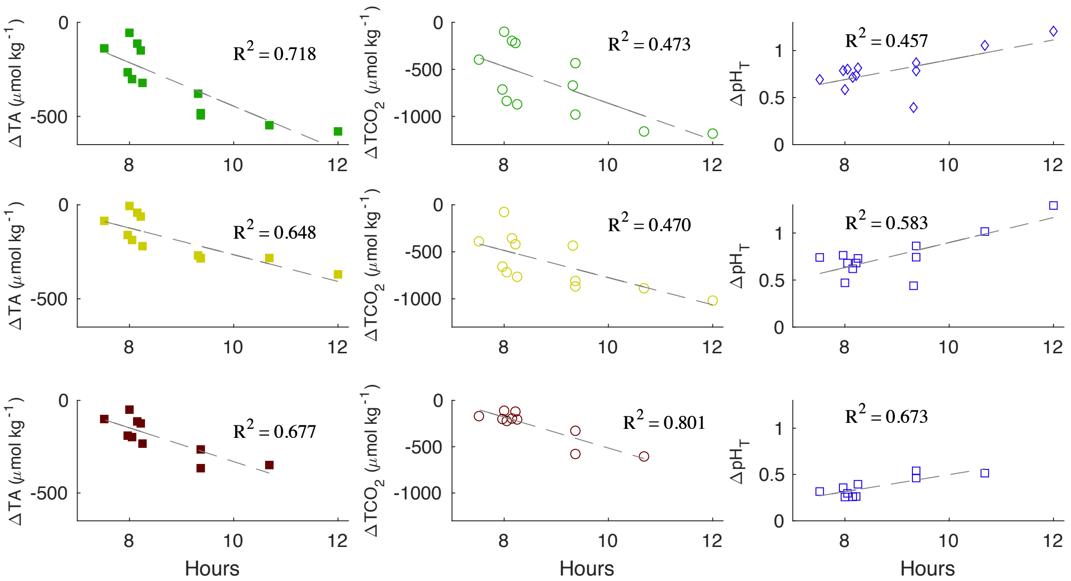

Figure S2. Changes in TA, TCO_2 and pH_T as a function of residence time (emersion period) where each point is the trajectory between measurements taken immediately after ebbing of the flood tide and before next flood. This was a total of 12 points for the DG (row 1) and PG (row 2) pools, and 10 points for the NG (row 3) pool due to evaporation of pool.

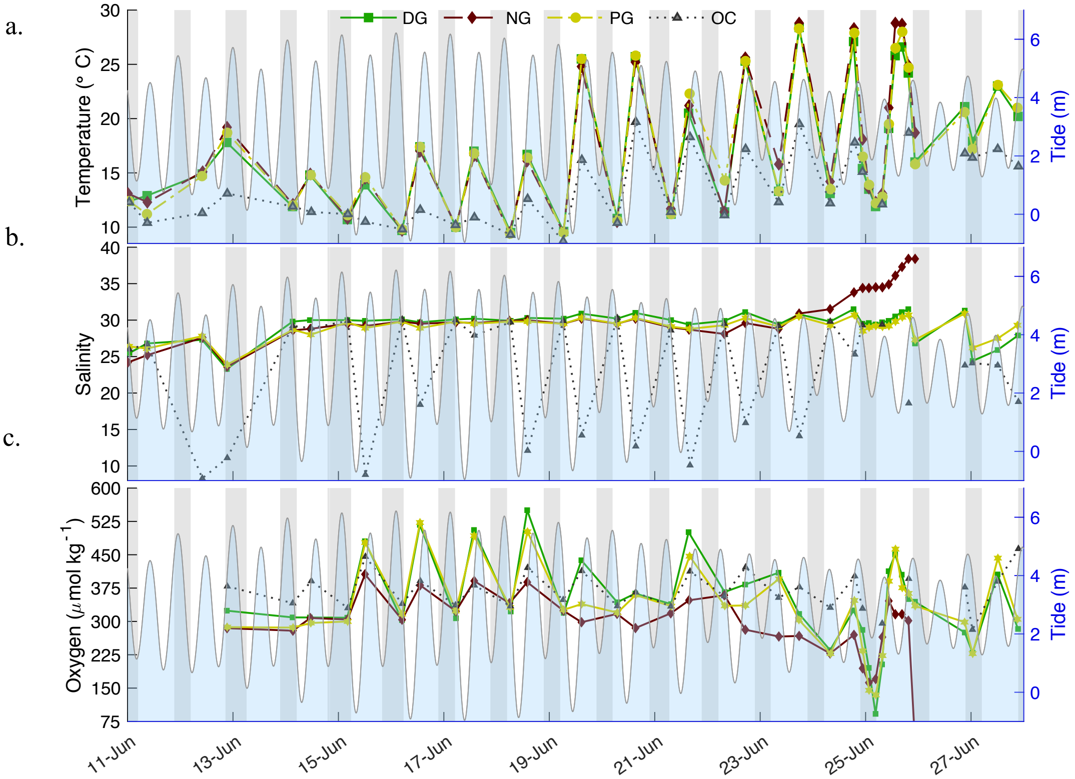

Figure S3. Timeseries of temperature (a), salinity (b), and oxygen (c) for dense grass (green squares), patchy grass (yellow circles), no grass (maroon diamonds), and ocean (grey triangles). Blue shaded region is predicted tidal height and grey shaded bars indicate nighttime PAR < 100 μmol photons m^{-2} s^{-1}.

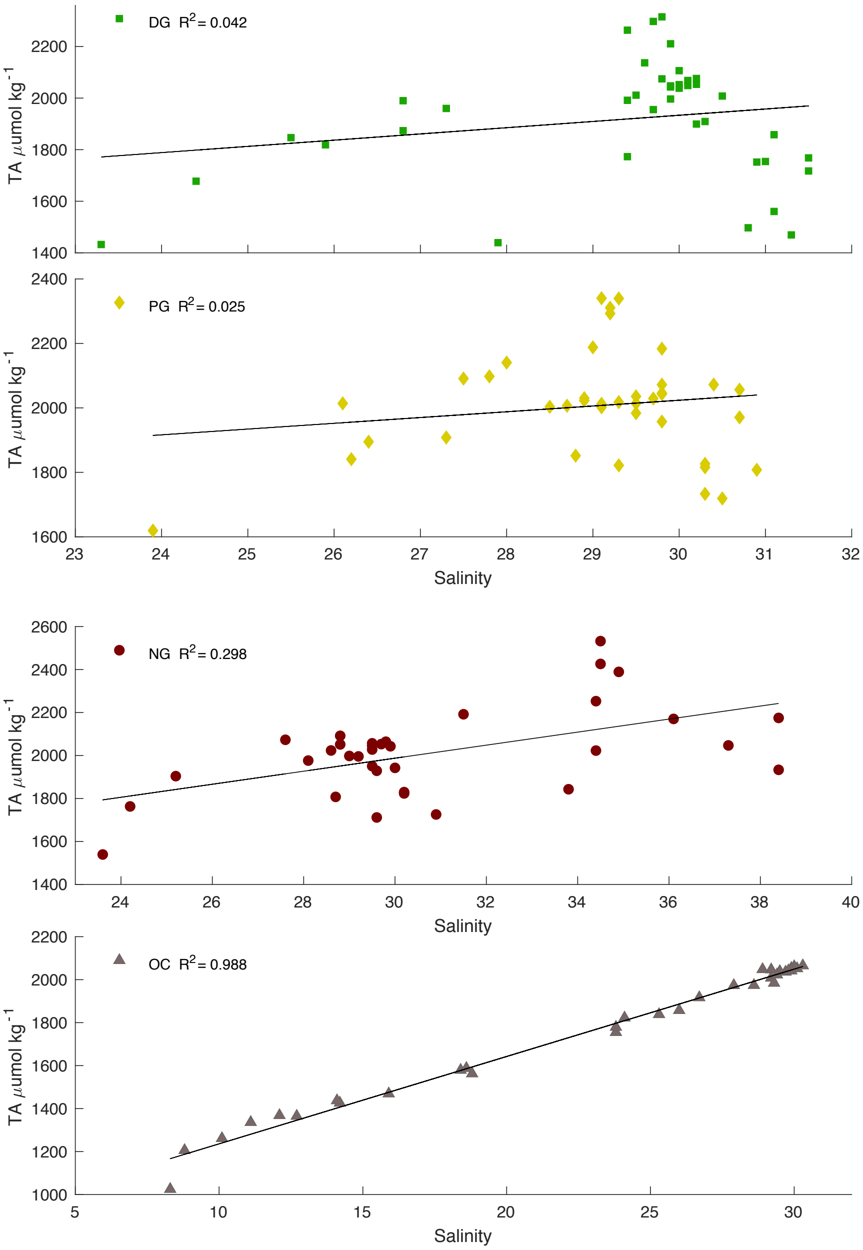

Figure S4. TA-salinity linear regressions for dense grass (DG), patchy grass (PG), no grass (NG), and ocean (OC). Note the different salinity scales for the NG and OC measurements.

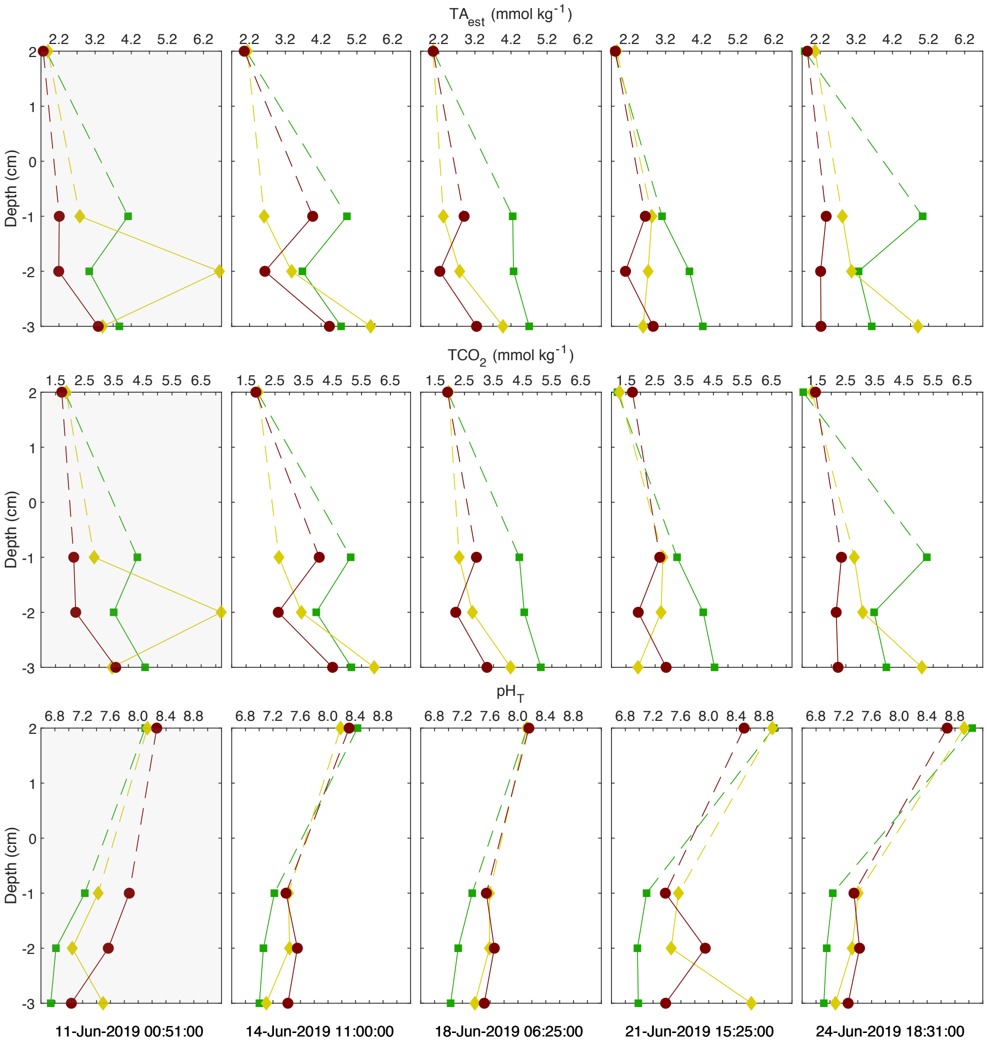

Figure S5. Porewater profiles of TA_{est} (top row), TCO_2 (middle row), and pH_T (bottom row) including overlying water at 1, 2, and 3 cm depth for dense grass (green squares), patchy grass (yellow diamonds), and no grass (maroon circles) pools. Carbonate alkalinity (TA_{est}) was estimated from pH_T and TCO_2 using CO2SYS. Shaded first column indicates nighttime measurements.

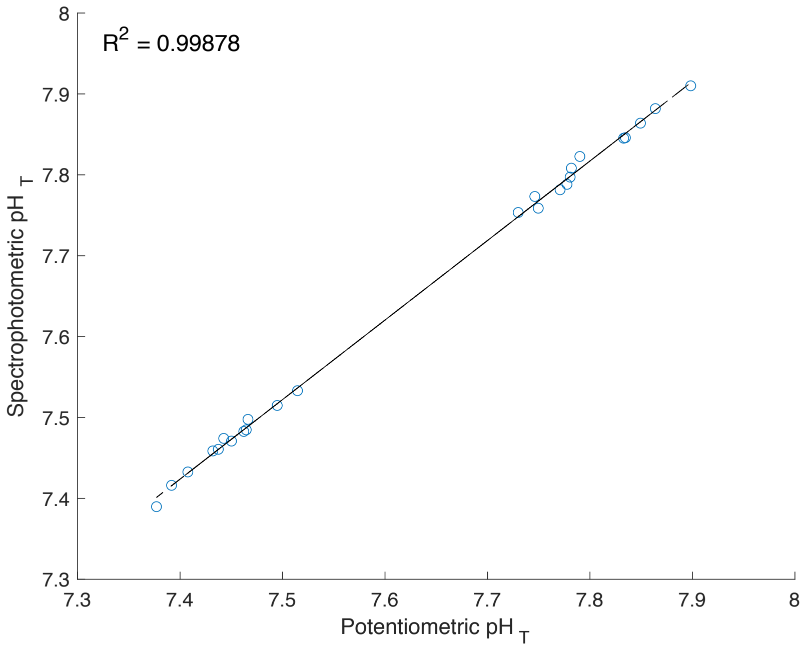

Figure S6. Linear regression between spectrophotometric pH and potentiometric pH.

Table S1. Changes in TA and TCO_2 (μmol kg^{-1} h^{-1}) for dense grass (DG), patchy grass (PG), and no grass (NG) pools calculated from individual discrete samples over the 17-d period from emersion to immersion time points (approximated linearly from 7 – 10 h periods).

	Range	
Pool	TA (μmol kg^{-1} h^{-1})	TCO_2 (μmol kg^{-1} h^{-1})
DG	6.96 – 52.79	12.50 – 108.6
PG	0.80 – 30.21	9.62 – 83.07
NG	6.23 – 39.07	13.94 – 56.67

Table S2. Nutrients for dense grass, patchy grass, and no grass pools collected every 3 h during 21 h sampling period.

	Dense grass				Patchy grass				No grass			
	NH_4^+	$NO_2^- + NO_3^-$	PO_4^{3-}	SiO_2	NH_4^+	$NO_2^- + NO_3^-$	PO_4^{3-}	SiO_2	NH_4^+	$NO_2^- + NO_3^-$	PO_4^{3-}	SiO_2
Date and Time	μmol L^{-1}	μmol L^{-1}	μmol L^{-1}	μmol L^{-1}	μmol L^{-1}	μmol L^{-1}	μmol L^{-1}	μmol L^{-1}	μmol L^{-1}	μmol L^{-1}	μmol L^{-1}	μmol L^{-1}
2019-06-24 22:30	9.75	0.35	0.62	1.48	2.03	2.54	0.00	10.22	0.00	0.00	0.62	19.45
2019-06-25 01:30	5.25	0.35	0.62	0.00	13.62	0.37	1.21	7.12	20.70	0.00	0.62	10.79
2019-06-25 04:30	7.82	0.00	0.62	0.00	2.67	0	1.21	12.34	1.38	0.00	0.62	7.62
2019-06-25 07:30	6.54	0.16	1.21	0.00	3.96	-0.2	0.62	9.85	7.82	0.00	0.62	2.71
2019-06-25 10:30	0.74	0.01	1.21	12.15	5.89	0.04	0.62	8.94	3.95	0.03	0.04	0.00
2019-06-25 13:30	12.97	0.00	0.62	0.00	0.00	0.08	0.62	3.99	11.69	0.16	0.62	0.00
2019-06-25 16:30	7.82	0.05	0.00	0.86	18.77	0.54	1.21	13.14	16.84	0.12	0.62	3.87
2019-06-25 19:30	7.82	0.00	0.62	19.29	8.47	0.38	0.62	23.39	3.96	0.00	0.62	10.88
2019-06-25 22:30	12.33	0.17	0.62	5.48	27.78	0.8	1.21	7.29	11.69	0.08	0.62	9.35
